# Impact of Four Weeks of TOGU Training on Neuromuscular Control and Golf Swing Performance

**DOI:** 10.3390/jfmk9040243

**Published:** 2024-11-19

**Authors:** Mohan Li, Caixian Ruan, Lin Zhang

**Affiliations:** Physical Education School, Shenzhen University, Shenzhen 518060, China; limohan6@outlook.com (M.L.); 19820009185@163.com (C.R.)

**Keywords:** TOGU, neuromuscular control ability, FMS, core strength training, golf

## Abstract

Purpose: To assess the impact of a four-week training program combining TOGU (a functional training system and equipment) Balanza and Dynair^®^ Ballkissen equipment on core strength, balance ability, and golf swing performance in golf athletes. Methods: The TOGU group participated in TOGU training three times weekly and regular golf skill training over four weeks. The control group only participated in regular golf skill training. The functional movement screening (FMS) assessment system modified the Clinical Test of Sensory Interaction on Balance (mCTSIB), and Unilateral Stance Tests (USTs) were used to assess neuromuscular control. Data are expressed as mean ± standard deviation (SD) and utilized the independent samples *t*-test and the paired *t*-test for statistical analysis. Results: (1) Following the four-week training, there was significant improvement of the TOGU group in the total score of FMS, notably in squats and in-line lunges (*p* < 0.05). (2) Significant reductions in COG sway velocity were observed: Foam-EO (−30.9%, *p* < 0.01) Firm-EC (−35.18%, *p* < 0.05) and Foam-EC (−36.78%, *p* < 0.001). UST also improved: L-EO (−34.39%, *p* < 0.001), L-EC (−29.92%, *p* < 0.001), R-EO (−48.67%, *p* < 0.01), and R-EC (−39.38%, *p* = 0.0857). (3) Club head speed (CHS) tests indicated significant enhancement (*p* < 0.01), improved ball speed (*p* < 0.001), driving distance (*p* = 0.0553), and hitting efficiency (*p* < 0.01). The control group showed no significant changes in all tests after four weeks of regular golf skill training. Conclusions: A TOGU-based golf core training program can significantly improve a golfers’ neuromuscular control, core stability, and coordination, and enhance their swing performance.

## 1. Introduction

Historically, golf has been considered a sport of technique and strategy, but recent research findings indicate that the movement predominantly engages the side of the body corresponding to the dominant hand during the swing, and prolonged repetition of such unilateral actions may lead to muscular imbalances, with one side of the body becoming significantly stronger and tenser than the other. This asymmetry can, in turn, restrict the range of motion, thereby escalating the risk of injury [1,2]. Previous studies have shown that core strength and balance training can improve the coordination of golf swings, increase club head speed, and prevent sports injuries induced by poor posture or weakened trunk strength [3]. However, golfers are subjected to relatively single-core muscle stability training with incomplete core training programs [4]. TOGU Balanza and Dynair^®^ Ballkissen (Germany) are two training equipment specifically crafted to enhance balance and core stability. Integrating the functionalities of both a balance board and fitness training tool, their purpose is to elevate the subject’s balance, core stability, and overall body strength. Similar devices have previously been used to enhance core strength in soccer training [5,6]. However, there has been no related research in the sport of golf, which also requires core strength enhancement.

The NeuroCom Balance Master system integrates advanced technology to provide precise evaluations of balance and postural control. Within this system, two pivotal sub-tests, namely the modified Clinical Test of Sensory Interaction on Balance (mCTSIB) and the Unilateral Stance Test (UST), play critical roles in assessing an individual’s stability and balance capabilities [7]. The mCTSIB evaluates static balance by measuring the center of pressure sway under varying sensory conditions, revealing postural stability across different scenarios. It involves standing on firm and foam surfaces with eyes open or closed, progressively challenging the sensory systems involved in balance maintenance [8]. The UST focuses on unilateral balance, testing each leg’s stability with eyes open and closed. It detects limb asymmetries and contributes to understanding the overall balance control and strength distribution in the lower limbs [9]. These tests offer a comprehensive evaluation of balance, facilitating tailored interventions for enhancing stability and improving overall functionality. Their combined use represents a thorough approach to balance assessment in clinical and research contexts.

Functional movement screening (FMS), which has emerged as an advanced method for assessing fundamental movement patterns in individuals, comprehensively examines flexibility and stability, identifying limitations, compensations, and asymmetries in body function [10]. Past research has shown that FMS scores are often positively correlated with core strength, making it a useful tool for evaluating both core strength and balance capabilities [11].

Research has shown that strength- and power-focused training programs offer benefits in several areas, such as club head speed and driving distance. Previous studies have revealed that high-level golfers with handicaps below zero exhibit statistically significant advantages over players with handicaps ranging from 0 to 20 in tests of hip strength, trunk strength, shoulder strength, shoulder and hip flexibility, and trunk flexibility—as well as static balance when tested with eyes open (*p* < 0.05) [12]. These results suggest that the FMS can be adopted to effectively evaluate the core strength and other qualities of golfers with a certain level of technical proficiency to assess their athletic performance. In addition, creating personalized programs based on players’ weaknesses identified through FMS scores can be beneficial. When combined, the advantages of FMS and corrective exercises can be maximized. This training method, combining personalized and core training approaches, can reduce injury risk, enhance strength and power, and improve flexibility while addressing overall bodily limitations. These improvements will also lead to improved driving distance, accuracy, and consistency [13].

In this study, we investigated the effects of a specially designed training scheme using TOGU equipment on the swing performance of golfers compared to singular golf skill training. Specifically, we ascertained whether a four-week integrated training program that incorporated TOGU exercises, along with core stability exercises, could enhance the swinging performance of sub-elite golfers. Performance improvements were measured through metrics such as club head speed, ball speed, strike efficiency, and driving distance. Additionally, FMS was executed to detect improvements in a golfer’s core strength and balance with our core training exercises.

## 2. Materials and Methods

### 2.1. Participants

**Figure 1:**
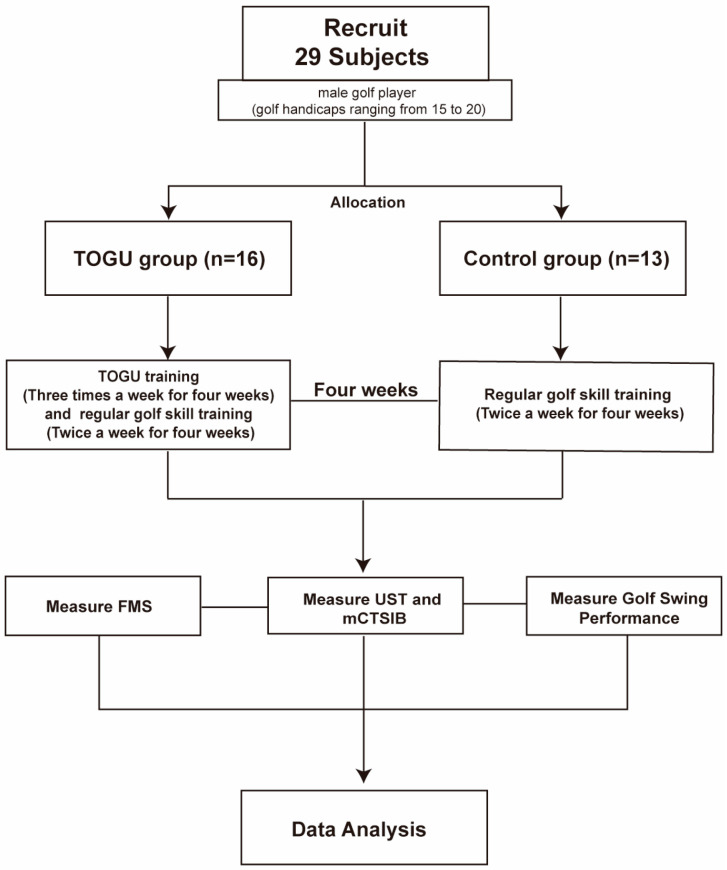
Experimental plan flowchart.

Twenty-nine right-handed men with golf handicaps ranging from 15 to 20 were recruited for this study. The recorded measures included mean age (21.06 ± 1.06 years), height (175.19 ± 5.14 cm), and weight (72.11 ± 10.04 kg). The twenty-nine participants were divided into two groups: the TOGU group and the control group. The control group consisted of 13 individuals who received regular golf skill training twice a week. The TOGU group, comprising 16 individuals, participated in the regular golf skill training sessions as well as additional core training (Figure 1). The subjects were asked to refrain from any physical activity training 24 h before testing. All participants were informed of the training plan and signed informed consent forms. Ethical approval for the commencement of the study was obtained beforehand from the Medical Ethics Committee of the Medical School, Shenzhen University (PN-202400082).

### 2.2. Exercise Program

**Figure 2:**
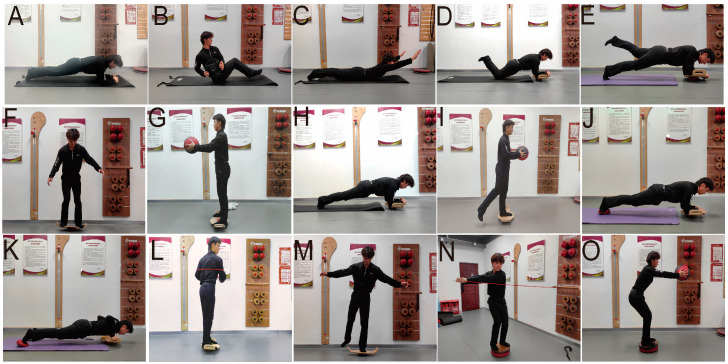
Depiction of exercise programs. (**A**): Plank. (**B**): Russian twist. (**C**): Prone back extensions. (**D**): Kneeling universal disc plank support. (**E**): Single-leg universal disc touch support on both sides. (**F**): Standing support on both feet with a universal disc. (**G**): Standing support on both feet with a universal disc while holding a medicine ball. (**H**): Plank exercise with a universal disc. (**I**): Single-leg standing support with a universal disc while holding a ball. (**J**): Plank exercise with a balance cushion. (**K**): Push-ups with a balance cushion. (**L**): Standing body rotation with rope. (**M**): Single-leg standing support with a universal disc. (**N**): Core anti-rotation while standing on a balance cushion. (**O**): Throwing medicine ball while standing on a balance cushion. The training movements generally involved the subject performing the entire golf swing motion with the power stick—including the setup, backswing, downswing, and follow-through—repeated in sequence.

Participants did not engage in any other training interventions besides the core training and regular golf skill training outlined in the study plan. The core training was divided into two phases (Table 1 and Figure 2). Both phases consisted of training sessions three times a week, each lasting 60 min. In addition to the primary components, each training session included a ten-minute warm-up, implemented both prior to the start and following the completion of the session. This consistent practice was aimed at promoting flexibility, preventing injuries, and facilitating recovery, thereby augmenting the training’s overall efficacy and safeguarding the participants’ well-being throughout the program [14].

### 2.3. Assessment of Neuromuscular Control Capability

**Figure 3:**
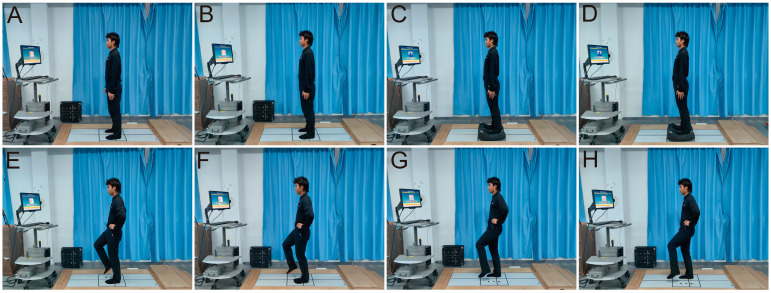
mCTSIB and UST subject-testing diagrams. (**A**). Firm surface standing with eyes open. (**B**). Firm surface standing with eyes closed. (**C**). Foam surface standing. (**D**). Foam surface standing with eyes closed. (**E**). Left foot standing with eyes open. (**F**). Left foot standing with eyes closed. (**G**). Right foot standing with eyes open. (**H**). Right foot standing with eyes closed.

To assess the balancing abilities of the subjects, we used the mCTSIB and USTs of the NeuroCom Balance Master (Balance and Mobility Therapy, Toledo, OH, USA). The mCTSIB tests involved standing under different conditions: (1) eyes open on a firm surface; (2) eyes closed on a firm surface; (3) eyes open on a foam surface; and (4) eyes closed on a foam surface (Figure 3A–D). A higher center-of-pressure sway velocity indicated poorer static balance. We collected data on the subjects’ balancing abilities both before and after the experimental intervention [15]. The UST is primarily designed to quantify one’s ability to maintain postural stability while standing on either the right or left leg, with eyes open or closed on a firm surface. The test comprised four tasks: (1) standing on the left leg with eyes open; (2) standing on the left leg with eyes closed; (3) standing on the right leg with eyes open; and (4) standing on the right leg with eyes closed (Figure 3E–H) [7].

### 2.4. Functional Movement Screen Testing

Prior to and following the intervention training, FMS testing was conducted on 29 participants, comprising seven exercise tests: deep squat (DS), hurdle step (HS), in-line lunge (ILL), shoulder mobility (SM), active straight-leg raise (ASLR), trunk stability push-up (TSPU), and rotary stability (RS). Each exercise was rated on a scale from 0 to 3, where 3 denoted the subject’s ability to act without compensation or pain. A score of 2 indicated completion without pain but with some degree of compensation or imperfection; 1 designated that the subject failed to execute the movement as instructed; and 0 was recorded if pain was experienced during the exercise or its associated clearance test, regardless of skill level [16]. Golf is classified as a unilaterally dominant sport, characterized by significant asymmetry in its execution. Among the seven tests that make up the FMS, five (HS, ILL, SM, ASLR, and RS) are bilaterally scored, enabling us to discern asymmetries in scores or discrepancies in performance between a subject’s sides [17].

### 2.5. Measurement of Swing Data

Club head speed, ball speed, and driving distance are commonly used indicators of golf performance [18]. We measured the swing performance of our subjects using the GCQuad launch monitor (Foresight Sports, San Diego, CA, USA) before and after interventional training. Prior to each measurement, we captured and analyzed a series of key indicators comprising club head speed to gauge power output, ball speed to assess energy transfer efficiency, a ball’s striking efficiency metric to reflect the actual effectiveness of power conversion, and ultimate total ball distance. These data points were compiled to profile the subjects’ swinging dynamics.

### 2.6. Statistical Analysis

We employed the quantitative approaches of mean ± SEM to depict the dispersion pattern and central tendency of data distribution and conducted two-way ANOVA (Analysis of Variance), using the independent samples design for comparing groups and the related samples design for assessing changes within the same group over time or under different conditions. We designated the differences between the two groups as statistically significant at *p* < 0.05. All data analyses and graphical renderings were conducted using GraphPad Prism version 10.2.2 (GraphPad Software, San Diego, CA, USA).

## 3. Results

### 3.1. Impact of a Four-Week TOGU Training Program on FMS Scores

**Figure 4:**
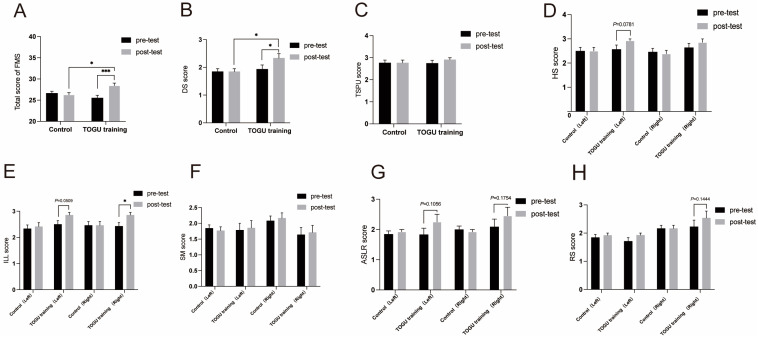
The impact of a four-week TOGU training program on FMS scores. (**A**). Total FMS score and differential analysis. (**B**). Deep squat score and differential analysis. (**C**). Trunk stability push-up score and differential analysis. (**D**). In-line lunge score and differential analysis. (**E**). Shoulder mobility score and differential analysis. (**F**). Active straight-leg raises score and differential analysis. (**G**). Hurdle step score and differential analysis. (**H**). Rotary stability score and differential analysis. Bar charts show the mean scores before and after the training program. Error bars represent standard error. * Indicates *p* < 0.05, *** indicates *p* < 0.001, as determined by two-way ANOVA. Significant differences are marked with asterisks above the connecting lines.

Following four weeks of core and balance training, we observed that the total FMS scores of the 16 participants of the TOGU group showed a highly significant improvement compared to their pre-training levels (*p* < 0.001, Figure 4A); and the total FMS score after training improved by 11.48%. Of the sub-tests, the deep squat demonstrated a significant improvement post-training (*p* < 0.05, Figure 4B), and the total score for the deep squat increased by 23.07%. However, there was no difference in trunk stability push-up performance before or after training (Figure 4C), with the total score increasing by only 6.06% after training. The left hurdle step revealed a significant improvement trend from pre-training levels (*p* = 0.0781 Figure 4D), with the score increased by 11.11%. The left in-line lunge demonstrated a clear improvement trend after the combined training (*p* = 0.0509, Figure 4E) and the right in-line lunge exhibited significant differences after training (*p* < 0.05, Figure 4E), with the total score of the left in-line lunge increasing by 14.28%, and the total score for the right in-line lunge improving by 17.64%. Neither side, with respect to shoulder mobility, showed a significant difference after training (*p* > 0.05, (Figure 4F). The active straight-leg raise on the left side showed a significant improvement trend compared to pre-training (*p* = 0.1056), with the total score increasing by 22.22%; while the right side displayed a similar significant improvement trend (*p* = 0.1754, Figure 4G), with the score rising by 9.3%. Right rotational stability demonstrated a significant improvement trend compared to pre-training (*p* = 0.1444), with the score augmented by 12.5%; whereas the left side did not show a difference in rotational stability (Figure 4H). In contrast, 13 participants in the control group showed no significant changes in any of the FMS items or the total score after four weeks of regular golf skill training (Figure 4A–H).

### 3.2. Effect of a Four-Week TOGU-Training Regimen on Core Strength and Balance Ability

**Figure 5:**
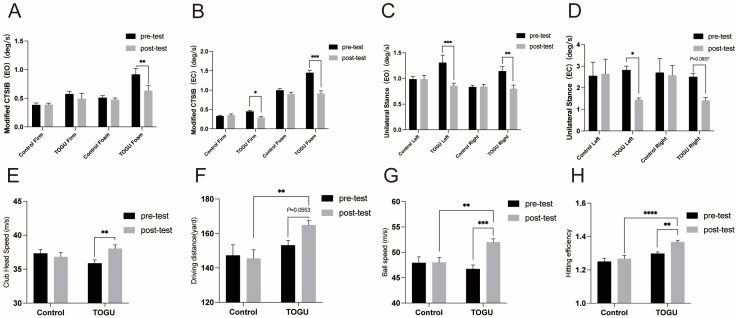
The effect of a four-week TOGU training regimen on core strength, balance ability, and golf swing performance. (**A**). mCTSIB eyes open differential analysis. (**B**). mCTSIB eyes close differential analysis. (**C**). UST eyes open differential analysis. (**D**). UST eyes close differential analysis. (**E**). Club head speed differential analysis. (**F**). Driving distance differential analysis. (**G**). Ball speed differential analysis. (**H**). Hitting efficiency differential analysis. Hitting efficiency is the ratio of ball speed to club head speed. Bar charts show the mean performance before and after the training program. Error bars represent standard error. * Indicates *p* < 0.05, ** indicates *p* < 0.01, *** indicates *p* < 0.001, and **** indicates *p* < 0.0001, as determined by two-way ANOVA. Significant differences are marked with asterisks above the connecting lines.

The data revealed that compared to before training, we noted significant improvements in COG sway velocity of the TOGU group in the mCTSIB test after training, while the control group did not show any significant changes. Specifically, there was a 30.9% reduction in the Foam-EO position (*p* < 0.01, Figure 5A), a 35.18% reduction in COG sway velocity in the Firm-EC condition following four weeks of training (*p* < 0.05, Figure 5B), and a 36.78% reduction in the Foam-EC position (*p* < 0.001, Figure 5B). We observed similar statistically significant improvements in UST, with a 34.39% diminution in COG sway velocity in the L-EO test (*p* < 0.001, Figure 5C), a 29.92% drop in the L-EC test (*p* < 0.05, Figure 5D), a 48.67% decline in the R-EO test (*p* < 0.01, Figure 5C), and a 39.38% decrease in the R-EC test (*p* = 0.0857, Figure 5D). The control group also showed no significant changes in the UST test (Figure 5C,D).

### 3.3. Effect of a Four-Week TOGU Training Regimen on Golf Swing Performance

Significant improvements in the TOGU group were observed in club head speed tests (*p* < 0.01, Figure 5E), which increased by 5.73%, and driving distance (*p* = 0.0553, Figure 5F) by 6.67%. The four-week training regimen also markedly enhanced ball speed (*p* < 0.001, Figure 5G) by 11.3%, and hitting efficiency (*p* < 0.01, Figure 5H) by 5.45%, while the control group did not show any significant changes (Figure 5E–H). Additionally, compared to simply conducting regular golf training, the training program that incorporated TOGU was more effective in increasing golfers’ driving distance (*p* < 0.01, Figure 5F), ball speed (*p* < 0.01, Figure 5G), and hitting efficiency (*p* < 0.0001, Figure 5H).

## 4. Discussion

The primary findings of our study revealed that compared to single regular golf skill training, the TOGU equipment combined with a training regimen significantly enhanced overall FMS scores and augmented performance in the mCTSIB and USTs by the 16 sub-elite golfers. Furthermore, the combination regimen led to a notable improvement in swing performance, including club head speed, ball speed, driving distance, and hitting efficiency.

Core strength serves as a bridge connecting the upper and lower limbs and is crucial for body stabilization and the effective transfer of power to the extremities. Our specially designed TOGU equipment yields a training regimen that differs markedly from conventional core strength training models both theoretically and practically. This innovative approach deepens and broadens the overall training philosophy. Traditional core exercises tend to focus on the isolation of muscle groups and static strength development, whereas the TOGU methodology accentuates the cultivation of dynamic balance and full-body synergy. By exploiting the inherent instability of TOGU equipment, the training emphasizes enhancements in neuromuscular control and proprioception among golfers. This dual emphasis on strength augmentation and the maintenance of motion balance and correctness thus ensures an efficient conversion of power into swing dynamics [19]. Moreover, this new training paradigm transcends the realm of simple strength augmentation by deeply integrating golf-specific movement patterns, achieving a smooth transition from fitness improvement to practical skill enhancement. In accordance with the usage of the TOGU equipment, the emphasis shifts from mere force accumulation to the timeliness of power output and precision in movement execution, thereby guaranteeing that augmented physical capacities are directly manifested in key performance indicators on the course—which include increased clubhead speed and longer driving distances [20]. In addition, personalization and specificity constitute additional hallmarks of this training system. Tailored to the individual physical conditions and technical nuances of each sub-elite golfer, our program allows for meticulous adjustments that ensure an alignment of training content with individual needs. This level of customization surpasses what standardized core training routines can offer. In general, the present approach not only addresses the specific physical demands of a golf swing but also facilitates efficient neuromuscular coordination, providing subjects with greater precision in swing control. In addition, the engaging and diverse nature of this training regimen contributes to increased participation and adherence, laying a solid foundation for long-term skill development [21].

The functional movement screen is a standardized assessment tool that is used to evaluate an individual’s fundamental movement patterns, body symmetry, stability, and flexibility, and has often been associated with improvements in athletic performance. This systematic assessment methodology not only provides a comprehensive pre- and post-training evaluation of functional movement capabilities, but also facilitates the identification of potential imbalances and allows the tracking of progress made through the intervention. These indices enhance our understanding of the efficacy of the training program by improving the overall movement quality and symmetry in participants. Investigators have previously demonstrated that eight weeks of isolated core training can marginally reduce variability in participants’ repeat club head speed and backspin, while slightly increasing sidespin variability, thereby contributing to a more consistent swing pattern [22]. Another study showed that a strengthened core enhances body control during the swing, enabling athletes to transmit force more efficiently to the club, resulting in increased club head velocity and ball speed [23]. Our study further corroborated these findings as evidenced by improved FMS scores, directly reflecting heightened bodily coordination and control among subjects executing the complex, multi-dimensional actions of a golf swing. Historically, the FMS has found application across various sports and in athlete training, rehabilitation medicine, and fitness domains. However, its utilization in researching enhancements specifically related to golf performance has been relatively underexplored in the realm of athlete training [24]. Following four weeks of our tailored training program, the subjects experienced a notable increase in their overall FMS score, with improvements observed in movement patterns such as the deep squat, in-line lunge, and active straight-leg raise. These enhancements are intimately linked to the hip joint flexibility, leg strength, and trunk stability demanded by a golf swing [25].

Additionally, the improvements noted in the subjects’ performance in mCTSIB and UST following the four-week training period also significantly contributed to the enhanced golf swing performance. Previous research has indicated that enhancements in closed-eye single-leg exercise performance are associated with improved dynamic balance and agility among athletes [26].

Previous studies by Wells and colleagues have highlighted the pivotal role of core strength and stability in golf swing efficiency, and reports by Weston and others emphasized the importance of core training for enhancing golf club head speed [27]. Our study reinforced these notions, as after four weeks of focused core strength and balance training, the subjects demonstrated significant improvements in their deep squat, in-line lunge, and active straight-leg raise scores. Scientists have previously associated increases in these scores with enhancements in the subjects’ dynamic-balance abilities and overall motor function [10], and professional golfers, in particular, tend to exhibit superior dynamic balance [28]. This may account, in part, for the observed improvements in the subjects’ swinging performance after the four-week training regimen, thereby validating the efficacy of the FMS scores in partially predicting golfers’ performance. Neither the total FMS score nor its individual components demonstrated statistically significant correlations with club head speed (CHS) in this study. In contrast, some reports suggest that an eight-week strength training regimen augments college golfers’ CHS by increasing muscular strength and power in both the upper and lower limbs [29]. Furthermore, Lewis and colleagues have documented significant correlations between squat jumps and seated medicine-ball throws with CHS in professional golfers, highlighting the role of these exercises in enhancing golf performance [30]. We attribute this discrepancy to the pre-existing level of physical competence among the subjects, which suggests a ceiling effect where further improvements (though potentially present) could not be statistically discerned post-intervention [31]. Moreover, given that all participants were right-handed, the left-sided scores tended to show greater improvement compared to the right in bilateral assessment tests (ASLR, HS, and RS). A statistically significant improvement was observed in the left HS test, whereas no such difference was detected on the right side, which likely was because the subjects had already reached a score of 3 on the HS for their right side prior to the intervention training. This observation is congruent with the acknowledged limitation of the FMS in finely discriminating among individuals who already possess a certain degree of athletic proficiency [32].

## 5. Conclusions

Our findings further solidify the positive impact of core strength and balance on golf performance. By combining TOGU with conventional sports training equipment through a distinctive training regimen, we have not only validated these theories but have also pioneered a novel and efficacious training pathway. This provides golfers and coaches alike with a scientifically grounded and systematic training strategy. Future studies may focus on the customization of training programs and explore methods to optimize training outcomes based on individual differences, with the goal of disseminating these benefits across a broader range of golf enthusiasts.

### Limitations

The research has the following limitations that need to be considered. To begin, the sample size of the experimental group needs to be enlarged to guarantee the dependability and validity of the experiment. Secondly, the intervention duration needs to be extended, and follow-up studies should be carried out regarding future sports performance and sports injuries. Thirdly, further exploration of the mechanism of neuromuscular control is necessary, for example, by starting from the perspective of brain science.

## Figures and Tables

**Table 1:** Core exercise program with TOGU Balanza.

weeks 1	**Exercise Program**	**(Sets × Repetitions)**
	Plank support	1 min × 4
	Russian twist	1 min × 4
	Prone back extensions	Achieve exhaustion × 4
	Kneeling universal disc plank support	1 min × 4
week 2–4	**Exercise Program**	**(Sets × Repetitions)**
	Single-leg universal disc touch support on both sides	1 min × 3
	Standing support on both feet with a universal disc	1 min × 4
	Standing support on both feet with a universal disc while holding a medicine ball	1 min × 4
	Plank support with universal disc	1 min × 4
	Single-leg standing support with a universal disc	1 min × 4
	Single-leg standing support with a universal disc while holding a ball	1 min × 4
	Push-up with balance cushion	1 min × 4
	Standing body rotation with rope	1 min × 4
	Single-leg standing support with a universal disc	1 min × 4
	Core anti-rotation while standing on a balance cushion	1 min × 2
	Throwing medicine ball while standing on a balance cushion	1 min × 2
	Balance air cushion standing power stick swinging	1 min × 4

## Data Availability

The data and materials this study is based on are available from the corresponding author, L.Z.
